# Zinc-positive and zinc-negative connections of the claustrum

**DOI:** 10.3389/fnsys.2014.00037

**Published:** 2014-03-18

**Authors:** Kathleen S. Rockland

**Affiliations:** ^1^Department of Anatomy and Neurobiology, Boston University School MedicineBoston, MA, USA; ^2^Cold Spring Harbor Laboratory, Cold Spring HarborNY, USA

**Keywords:** amygdala, epilepsy, feedback, layer 1, reuniens

Three features often mentioned as characteristic of the claustrum are its widespread connections with cortical areas, the reciprocity of these connections in general, and the origin of cortico-claustral connections from a distinctive subtype of layer 6 pyramidal cells (Sherk, 1986; Katz, 1987; Tanne-Gariepy et al., 2002; Crick and Koch, 2005; Smythies et al., 2012). Another feature, often overlooked, is that a proportion of claustral-cortical neurons use synaptic zinc and that zinc+ terminations are moderately dense in the claustrum. This article will summarize data about zinc and the claustrum and present the case that cortico-claustral neurons might also be zinc-positive (Zn+). I conclude with comments on the likely implications for claustral identity and function.

## Zinc-containing neurons

There are several excellent reviews on the importance of synaptic zinc in the central nervous system (Frederickson et al., 2000, 2005; Nakashima and Dyck, 2009; Sensi et al., 2009). In brief, Zn+ neurons are predominantly in the cerebral cortex and amygdala. Zn+ neurons (i.e., those in which zinc is highly concentrated in the synaptic vesicles) are a subset of glutamatergic neurons, exclusive of corticothalamic. The projections between thalamus and cortex are zinc-negative (Zn−). Zinc is considered an activity- and calcium-dependent neuromodulator of excitation and as being important in synaptic plasticity. It interacts with a wide range of zinc-sensitive postsynaptic membrane targets (see Table 1 in Frederickson et al., 2005 and Figure 2 in Sensi et al., 2009). In general, Zn+ neurons have been preferentially associated with limbic projections; and the hippocampal mossy fibers are well-known to have a high concentration of synaptic zinc.

The brainwide distribution of Zn+ neurons is demonstrated by intraperitoneal injection of sodium selenite, which produces a zinc–selenium precipitate that is retrogradely transported from axon terminals to cell bodies of origin (e.g., Brown and Dyck, 2004). Focal intra-cerebral injections of sodium selenite are used to retrogradely label target-specific projection neurons. Terminations are visualized by modifications of the classic Timm stain.

## Claustral-cortical projections

Zinc–selenium histochemistry reveals a subset of Zn+ neurons in the mouse claustrum (Brown and Dyck, 2004); and focal injections of sodium selenite in different cortical areas in rodent directly demonstrate a subset of Zn+ cortically projecting neurons in the claustrum. These are reported as sparse or moderate for projections, respectively, to visual and barrel cortex, but more abundant for those to frontal and orbital cortical areas (Garrett et al., 1992; Casanovas-Aquilar et al., 1998; Brown and Dyck, 2005). Experiments using standard retrograde tracers report a small proportion of double labeled neurons projecting to two different cortical areas (in rat: Li et al., 1986; in cat: Clasca et al., 1992). Whether these are Zn+ or not is unknown.

The claustrum also sends projections to the amygydala (for monkey: Stefannacci and Amaral, 2000), to parts of the subicular complex (Witter et al., 1988; Zhang et al., 2013), and to nucleus reuniens (for rat: McKenna and Vertes, 2004). There are no data as to the proportion of claustral-amygdala or claustral-hippocampal neurons that might be Zn+; and claustral-thalamic projections can be assumed as Zn−.

Claustral-cortical projections labeled with standard anterograde tracers terminate in layers 1–4 and 6 (Clasca et al., 1992; da Costa et al., 2010). This partially coincides with the pattern of Zn+ terminations, which are elevated in layers 1b, 2, 3, and 5/6, depending on the cortical area. As layer 4 is relatively Zn−, claustral-cortical terminations in this layer can be inferred to originate from a separate subset of Zn− neurons in the claustrum. The proportion of Zn+ terminations may be taken to vary depending on the species and projection system. For claustral-cortical projections, an initial guess might be 25–50% as being Zn+, largely based on the density of Zn+ neurons retrogradely labeled in the claustrum following focal injections of sodium selenite in cortical areas in rodents.

By comparison, cortical feedback projections from monkey temporal cortex have been shown to consist of a mix of Zn+ and Zn− components by cortical injections of sodium selenite. In confirmation, injections of the anterograde tracer BDA in area TE were coupled with a terminal intravenous injection of sodium sulfide to precipitate Zn+ terminations. Subsequent ultrastructural inspection of BDA-labeled terminations in areas targeted by TE neurons (V1, V4, TEO, and the depth of the superior temporal sulcus) revealed about one-third of the synapses as Zn+, except for a higher proportion in V1 (four of five identified synapses; Ichinohe et al., 2010). As a second comparison, projections from the basolateral amygdala to medial prefrontal cortex were all found to be Zn+ in the monkey (Miyashita et al., 2007), although only 35% of the amygdalo-cortical terminations were Zn+ in rats (Cunningham et al., 2007).

## Cortico-claustral neurons

Layer 6 contains a mixed population of pyramidal neurons, of which the four major groups are intrinsically projecting, and extrinsically projecting to the thalamus, to other cortical areas, and to the claustrum (Briggs, 2010; Thomson, 2010). Corticothalamic neurons are Zn−; and feedback cortically projecting neurons in layer 6 are intermixed Zn+ and Zn− (for monkey: Ichinohe et al., 2010). There have been no appropriate injections in the claustrum to determine directly whether any cortico-claustral projecting neurons are Zn+, but this possibility is supported by indirect evidence, as discussed next.

First, as noted above, Zn+ terminations are moderately dense in the claustrum (rat: Perez-Clausell, 1996; Valente et al., 2002; monkey: Figure 1 in Ichinohe and Rockland, 2005a; Figure 10 in Miyashita et al., 2007). Zn+ cortico-claustral neurons are one of three possible sources of the Zn+ terminations in the claustrum. Another is the claustrum itself, since it has both Zn+ neurons (Brown and Dyck, 2004) and widespread intrinsic connections (Smith and Alloway, 2010). The amygdala is a third possible source. Several claustral projecting subnuclei in the amygdala contain Zn+ neurons, demonstrated by intraperitoneal (Brown and Dyck, 2004) or focal injections of sodium selenite in cortical areas (for the rat: Majak et al., 2002; for monkey: Ichinohe and Rockland, 2005b). There are projections from midline thalamus to the claustrum (Vertes et al., 2006), but like almost all thalamic projections (except those from anterior dorsal thalamus to the subiculum), these can be considered as zinc-negative.

A second, indirect line of evidence is the dendritic morphology of pyramidal neurons in layer 6 (Katz, 1987; Ojima et al., 1992; Olsen et al., 2012; and reviewed in Briggs, 2010; Thomson, 2010). Cortico-thalamic neurons have short, thin apical dendrites typically not extending much above layer 4. At least a subset of cortical feedback projecting neurons also have short, non-tufted apical dendrites (for monkey: Lund et al., 1981; Figure 9 in Rockland, 1994; Berezovskii et al., 2012). Cortico-claustral neurons have nontufted apical dendrites ascending to layer 1 (Katz, 1987). Of these three groups, cortico-thalamic neurons can be assumed to be Zn−. Cortico-cortical neurons, in monkey, are a mix of Zn+ and Zn−, as noted above. An interesting possibility is that some cortico-claustral neurons, which have a nontufted apical dendrite (even though this appears to ascend more superficially than cortical neurons), are Zn+. The proportion of layer 6 neurons with long apical dendrites (i.e., putative cortico-claustral) is likely to be area and species specific. From intracellular fills, these are reported as unusually abundant—almost 40% of the filled neurons—in layer 6 of rat medial prefrontal cortex, although the projectional identity is unknown (Van Aerde and Feldmeyer, 2013).

If cortico-claustral neurons, or a subset of these, are Zn+, we can further speculate whether individual neurons might send collaterals to cortical areas and the claustrum. There are so far no relevant data for cortico-claustal neurons, either from double retrograde tracers or intercellular labeling; and this possibility waits for future investigations.

What can we conclude about the claustrum as part of a Zn+ associational system? One clear point is that claustral-cortical neurons are a mixed population, of Zn+ and Zn− neurons, and are presumably functionally mixed as well. Less clear is the specific role or roles of zinc in the claustrum. In general, synaptic Zn is associated with activity-dependent plasticity (reviewed in Frederickson et al., 2005; Nakashima and Dyck, 2009). Consistent with plasticity effects, a sizeable proportion of claustral-cortical synapses are perforated (~33% in cat visual cortex; da Costa et al., 2010). By comparison, 27% of amygdalo-cortical terminations (putatively Zn+ but neurochemically uncharacterized) were identified as perforated in temporal cortex, 39% in visual cortex (for monkey: Freese and Amaral, 2006), and ~25% of those in orbitofrontal (identified as Zn+ for monkey: Miyashita et al., 2007). Perforated synapses are specifically implicated in memory-related plasticity (Calverley and Jones, 1990; Hara et al., 2012).

Alterations in the regulation of zinc release, either as protective or harmful, have been associated with epilepsy, among other neuropathological disorders (reviewed in Paoletti et al., 2009). The well-established susceptibility of the claustrum to kindling and its implication with generalized seizures may thus be related to the presence of zinc in intrinsic claustro-claustral connections and/or in the connections between the claustrum and amygdala or the claustrum and cortex. A recent study, concerned with the role of zinc homeostasis in epileptogenicity, found that epilepsy-resistant rat strains had significantly lower levels of synaptic zinc as compared to epilepsy-prone strains (Flynn et al., 2007).

## Claustrum as cortical?

The identification of the claustrum, as cortical or striatal, has generated considerable discussion. On developmental grounds, the claustrum has been considered (1) as a derivative of the insula, with a pallial origin; (2) as derived from the ganglionic eminence along with the basal ganglia; or (3) as having both a pallial and subpallial derivation (reviewed in Inda et al., 2009; Pirone et al., 2012). Gene expression studies show the claustrum as having pallial markers, like the amygdala but unlike striatal structures (Miyashita et al., 2005; Pirone et al., 2012); and the claustrum is consistently reported as expressing genes in common with cortical areas (Miyashita et al., 2005; Mathur et al., 2009; Watakabe et al., under review). From a somewhat different perspective, chandelier cells, a specific type of cortical interneuron, are found in both the claustrum and amygdala, but not striatum (Inda et al., 2009). From the perspective of zinc, both the claustrum and basal ganglia have moderate levels of Zn+ terminations, at least in part of a cortical origin; but Zn+ neurons do not occur in the striatum (Frederickson et al., 2000). The existence of Zn+ neurons in the claustrum is consistent with a cortical association, but is a feature shared as well with the amygdala.

## Claustral function?

One of the ideas consistently put forth for claustral function is that it is concerned with multisensory integration (Sherk, 1986; Edelstein and Denaro, 2004; Crick and Koch, 2005). This is consistent with its pattern of widespread connectivity, although physiological recording in alert monkeys have identified distinct claustral zones comprised of unimodal, not multimodal, neurons associated with the auditory and visual modalities (Remedios et al., 2010).

Reciprocal and widespread connectivity architecture, often seen as indicating an integrative role (Tanne-Gariepy et al., 2002; Crick and Koch, 2005), is not anatomically unique to the claustrum, but applies to other structures as well; for example, the amygdala and midline thalamus. Thus, it is not yet clear that this connectivity architecture in itself is strong support for a distinctively integrative role. Continuing work in rat, in fact, has concluded that while the efferent connectivity of the claustrum might well subserve interhemispheric coordination of motor and somatosensory whisker representations, its role as an integrator of somesthetic and motor information is less likely, since there are no projections from the somatosensory whisker representation to the claustrum (Smith et al., 2012).

Worth noting is that claustral-cortical projections terminate in both layers 1 and 4, presumably from separate subpopulations, given that Zn+ terminations are dense in layer 1 and very sparse in layer 4. Layer 1 and layer 4 terminations are also spatially dissociable to some extent in that those in layer 1 are typically widely divergent, in contrast with the more topographically organized termination systems in layer 4. Amygdalo-cortical projections to layer 1 are widely divergent (Freese and Amaral, 2006), as are thalamo-cortical (Rubio-Garrido et al., 2009), and cortical feedback (Rockland, 1994). Widespread terminations in layer 1 might contribute to the generation of synchronized oscillations, another role associated with the claustrum (Smythies et al., 2012), although more data are needed specifically concerning neurons postsynaptic to claustral inputs. In particular, is there a neuron-to-neuron reciprocity with claustral projections targeting cortico-claustral neurons?

The architecture that emerges is not so much structure-to-structure reciprocity, as a wider constellation of closely interconnected networks; namely, claustral-cortex-amygdala (Zn+ or mixed), claustral-hippocampus-amygdala (Zn+ or mixed), claustral-reuniens-cortex (Zn−), possibly hippocampus-reuniens-claustrum (Zn−), among others.

## Summary

The importance of synaptic zinc for claustral connections has been largely overlooked, despite abundant evidence of Zn+ inputs and outputs. Synaptic zinc has been associated with activity-driven plasticity; and one might propose that the functional role of zinc for the claustrum is “similar” to that of zinc as used by the basolateral amygdala and feedback cortical connections from layer 6. More immediately, a practical consequence is that the wide range of manipulations targeting Zn+ terminations and Zn+ neurons (reviewed in Nakashima and Dyck, 2009) offer new tools to probe claustral organization and function. Potential approaches might include comparisons across species and mouse lines, across developmental stages, or in different environmental or pathological conditions.
